# Death of unknown cause? Post-mortem diagnosis of fulminant course of an EBV-associated secondary hemophagocytic lymphohistiocytosis

**DOI:** 10.1007/s12254-021-00701-9

**Published:** 2021-04-01

**Authors:** Josia Fauser, Stefan Köck, Eberhard Gunsilius, Andreas Chott, Andreas Peer, Adelheid Ditlbacher, Gernot Fritsche, Michael Joannidis, Dominik Wolf, Andreas Pircher

**Affiliations:** 1 Department of Hematology and Oncology, Internal Medicine V, Medical University of Innsbruck, Anichstrasse 35, 6020 Innsbruck, Austria; 2 Institute of Pathology and Microbiology, Wilheminenspital, Vienna, Austria; 3 Division of Intensive Care and Emergency Medicine, Department of Internal Medicine, Medical University of Innsbruck, Innsbruck, Austria; 4 Department of Internal Medicine II, Medical University of Innsbruck, Innsbruck, Austria

**Keywords:** Fever of unknown origin, Hemophagocytic lymphohistiocytosis, Hemophagocytosis, HScore, Epstein–Barr virus

## Abstract

HLH is a life-threatening disease, which is characterized by a dysregulated immune response with uncontrolled T cell and macrophage activation. The often fulminant course of the disease needs a fast diagnostic work-up to initiate as soon as possible the appropriate therapy. We present herein the case of a 71-year-old patient with rapidly progressive hyperinflammatory syndrome, which post mortem resulted in the diagnosis of EBV-associated HLH. With this case report, we intend to highlight the relevance of the HScore in the diagnosis of HLH, to create a greater awareness for EBV as a trigger of HLH, and to demonstrate the importance of treating EBV-associated HLH as early as possible.

## Case presentation

A 71-year-old man was transferred from a peripheral hospital to our intensive care unit (ICU) for further work up of a hyperinflammatory syndrome (CRP 6.3 mg/dl [< 0.5 mg/dl]; IL‑6 63.5 ng/l; LDH 679 U/l; ferritin 2587 µg/l; sIL-2R > 20 ng/ml) with recurrent fever episodes of unknown origin for 7 days and hemodynamic instability.

Initially the patient was admitted to a peripheral hospital due to physical weakness, nausea and hypotension. Pre-existing medical conditions included dilated cardiomyopathy with atrial fibrillation and arterial hypertension, chronic renal failure and type 2 diabetes mellitus, but did not include immunosuppression. Blood tests revealed thrombocytopenia (52 g/l), hypercalcemia (3.44 mmol/l), and hyperlactatemia (55.9 mg/dl) without acidosis. There was also evidence of inflammation (CRP 3.5 mg/dl, ferritin 1125 ng/ml) without a clear infectious focus. The patient was cardiorespiratory and hemodynamic stable without clinical or echocardiographic signs of cardiac decompensation but showed already impaired liver (bilirubin 2.21 mg/dl) and kidney function (GFR 26 ml/min). Despite a broad anti-infective therapy with piperacillin/tazobactam, moxifloxacin and acyclovir, systemic inflammation progressed rapidly, which required the transfer of the patient to our ICU.

At admission to our department, the patient was in reduced general condition (Eastern Cooperative Oncology Group–Performance Status of 4) and therefore already required support with noradrenaline due to hemodynamic instability. Furthermore, initial laboratory tests confirmed the impaired liver (bilirubin 12.27 mg/dl; INR 1.3; albumin 2.3 g/dl) and kidney function (GFR 15 ml/min).

Blood tests for clarification of hypercalcemia, revealed suppressed parathyroid hormone (PTH 1.2 ng/l) and elevated calcitriol (147 ng/l), but normal levels of serum phosphate (1.12 mmol/l) and urinary calcium (4.46 mmol/l). The patient was administered denosumab once to adjust the elevated calcium levels. Since serum electrophoresis excluded paraproteinemia and the FLC ratio proved normal, the initial suspicion of multiple myeloma was withdrawn. The computed tomography (CT) body scan revealed no osteolysis, but showed extensive lymphadenopathy (supraclavicular, mediastinal, abdominal, retroperitoneal) and splenomegaly, thus, raising the suspicion of cancer. A bone marrow aspiration revealed a punctio sicca. For further clarification, a lymph node was extirpated; however, thereafter the clinical status of the patient rapidly deteriorated. Infectiology work up showed a negative plasma CMV-PCR; however, EBV DNA count (2309 IU/ml) was significantly elevated.

**Fig. 1:**
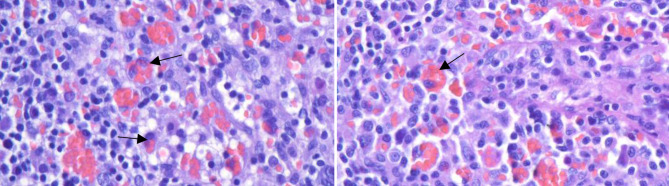
Representative image from the removed cervical lymph node showing erythrophagocytosis (*arrows*)

Due to the suspicion of a hyperinflammatory syndrome, the HScore was calculated (Table 1). The HScore revealed 208 points (ferritin 2587 µg/l, triglycerides 2.61 mmol/l, fibrinogen 196 mg/dl, bicytopenia, splenomegaly, fever) leading to the working hypothesis of a secondary hemophagocytic lymphohistiocytosis (sHLH) triggered by lymphoma or EBV reactivation (Table 2). Due to the high clinical urgency, immediate treatment with high-dose methylprednisolone was initiated. Despite the immunosuppressive therapy, continuous hemofiltration and catecholamine administration, hemodynamic instability and metabolic acidosis rapidly aggravated (pH 7.05; lactate 179 mg/dl). The patient died 2 days after admission to our ICU. The histology of the extirpated lymph node showed preserved lymph node architecture and nondestructive infiltration of large atypical mononuclear and multinucleated cells, which were positive in the EBER in situ hybridization. In addition, numerous T cells and CD68R+ histiocytic cell forms showing massive hemophagocytosis were evident (Fig. 1).

**Table 1:** The HScore helps in the diagnosis of secondary HLH. The probability of a HLH is calculated from the sum of the points

Parameter	No. of points (criteria for scoring)	Patient’s score at admission
Known underlying immunosuppression	0 (no) or 18 (yes)	0
Temperature (°C)	0 (< 38.4), 33 (38.4–39.4), or 49 (> 39.4)	33
Organomegaly	0 (no), 23 (hepatomegaly or splenomegaly), or 38 (hepatomegaly and splenomegaly)	23
No. of cytopenias	0 (1 lineage), 24 (2 lineages), or 34 (3 lineages)	24
Ferritin (ng/ml)	0 (< 2000), 35 (2000–6000), or 50 (> 6000)	35
Triglyceride (mmol/l)	0 (< 1.5), 44 (1.5–4), or 64 (> 4)	44
Fibrinogen (g/l)	0 (> 2.5) or 30 (≤ 2.5)	30
Serum glutamic oxaloacetic transaminase (IU/l)	0 (< 30) or 19 (≥ 30)	19
Hemophagocytosis features on bone marrow aspirate	0 (no) or 35 (yes)	0
TOTAL	–	208

**Table 2:** Fact sheet

Relevant diagnostic findings of our patient	
Symptoms	Fever attacks, hypotension, nausea
HScore (Table 1)	208 points (88–93% probability of the presence of HLH)
CT scan	Splenomegaly, generalized lymphadenopathy
EBV DNA (PCR)	2309 IU/ml

## Diagnosis

Post-mortem, histological examination of the lymph node revealed the diagnosis of EBV-associated HLH.

## Discussion

This case shows the fulminant course of sHLH due to EBV reactivation. HLH is a rare, yet often life-threatening disease pattern, from the group of histiocytoses [1] and was first described in 1939 [2]. It is characterized by a dysregulated immune response with uncontrolled T cell and macrophage activation leading to massive cytokine release [3]. HLH can be divided into two groups, a primary, genetic and a secondary, acquired form. The primary form often affects children and young adults and results from genetic defects impairing the cytolytic function of CTL and/or NK cells [4] or the regulation of the inflammasome [5]. Secondary HLH can be triggered by infectious and noninfectious events. While viral infections (EBV, CMV, influenza, COVID-19) are the most common triggers of sHLH, other causes subsume lymphomas, rheumatological diseases or immunosuppression [6, 7]. It is often difficult to differentiate between HLH and sepsis clinically. Recurrent fever attacks and hepatosplenomegaly are characteristic but unspecific. Hyperferritinemia, often accompanied by bicytopenia, is a strong indication for HLH. Moreover, the diagnostic criteria of the HLH 2004 Guidelines or the HScore are helpful for the evaluation of HLH [8]. The advantage of the HScore compared with the HLH2004 criteria is that most parameters are quick and easy to collect, which may allow earlier diagnosis.

The cause of hypercalcemia in our patient remains unclear, moreover there are no reports of hypercalcemia in the context of HLH. In our case, the constellation of suppressed parathyroid hormone and elevated calcitriol in the absence of osteolysis suggests an ectopic production of calcitriol. One possibility is extrarenal synthesis of calcitriol by the 1α-hydroxylase in activated macrophages as known from sarcoidosis [9, 10]. Furthermore, a calcitriol production by lymphoma cells is described [11]. A paraneoplastic synthesis of parathyroid hormone related peptide (PTHrP) seems unlikely since these cases are usually associated with low or normal calcitriol serum levels [12, 13].

As exemplified in this case, the often life-threatening clinical picture of sHLH requires a quick diagnosis in order to initiate causal therapy, depending on the corresponding trigger. Since in our case, the disease was already in an advanced stage at admission, the patient died within 2 days and the final diagnosis could only be made postmortem. Another diagnostic challenge is that EBV can directly cause HLH but can also promote the progression of other types of HLH [14]. The prognosis of EBV-HLH remains poor, with a 1-year mortality rate of 75% in a study comprising 61 patients in 2015 [15]. In addition to EBV association, age > 45 years, low-platelet counts (< 35 G/L), hyperferritinemia (> 20,000 ng/ml) and missing/lack of response after 8 weeks are associated with negative prognosis [16]. Since there is no effective antiviral therapy against EBV, a causal treatment is not possible [17]. The therapy of EBV-associated HLH is based on the HLH-94/2004 regime. It consists of the administration of etoposide, a high dose of steroids, and at a later stage of treatment, cyclosporine A. In the case of central nervous system involvement, an intrathecal administration of methotrexate must be evaluated [18, 19]. An immediate start of therapy after diagnosis, especially with etoposide, is prognostically, highly relevant [20, 21]. Since EBV replicates in B‑cells, a further therapeutic approach represents the use of rituximab for B‑cell depletion and consequently the reduction of the EBV viral load [22]. Since it has been shown that in EBV-HLH T‑cells are also infected, rituximab is only recommended in combination with etoposide and dexamethasone [23, 24]. An alternative to the HLH94/2004 regimen is L‑DEP (liposomal doxorubicin, etoposide and high-dose methylprednisolone in combination with PEG-asparaginase), which has shown high response rates not only as initial but also as salvage therapy [25]. The HLH-94/2004 regimen is most commonly used and shows an initial response, but nonetheless, patients often suffer a relapse. Consequently, several therapies are currently tested in a refractory/relapsed setting. A promising analysis of 7 patients with EBV-HLH who were treated with nivolumab every 3 weeks showed that 5 of these patients had CR [26]. Other compounds in clinical trials are the JAK 1/2 inhibitor ruxolitinib [27] and the IFNγ-neutralizing antibody emapalumab [28], which has already received FDA approval for primary HLH. The ultimate treatment of refractory EBV-HLH remains allogeneic stem cell transplantation [29].

In our case, it must be questioned whether there was a window of opportunity in which the patient might have been saved by immediate therapy with etoposide, rituximab, dexamethasone, intravenous immunoglobulins, and full ICU support. At admission to our ICU, the HScore was already 208 points, corresponding to an 88–93% probability of the presence of HLH. In the HScore, the histological evidence of hemophagocytosis, which was not yet present in our patient at the time of admission, plays a minor role. Nevertheless, at that time it was unclear whether the hyperinflammation and lymphadenopathy was caused by a lymphoma or indeed by an EBV infection. It needs to be discussed whether, due to the life-threatening situation, histological evidence of hemophagocytosis and its genesis should be foregone in favor of increasing the probability of survival by starting therapy immediately. It should be noted, however, that even with immediate therapy, our patient’s prognosis would have been poor because of the high lethality of EBV-associated HLH, the already incipient multiple organ failure, and the multiple comorbidities.

## Conclusion

It is important to consider the possibility of sHLH and to calculate the HScore in patients with recurrent fever attacks without a clear focus of infection and rapid clinical deterioration. In generalized lymphadenopathy, EBV reactivation as the trigger of HLH should always be excluded. If the diagnosis of EBV-associated HLH is highly probable or confirmed, etoposide-containing therapy must be initiated immediately.
